# Recent climate warming drives ecological change in a remote high-Arctic lake

**DOI:** 10.1038/s41598-018-25148-7

**Published:** 2018-05-01

**Authors:** Lineke Woelders, Jan T. M. Lenaerts, Kimberley Hagemans, Keechy Akkerman, Thomas B. van Hoof, Wim Z. Hoek

**Affiliations:** 1 Department of Earth and Environmental Sciences, K.U. Leuven, Celestijnenlaan 200E, 3001 Leuven, Belgium; 2 Department of Atmospheric and Oceanic Sciences, University of Colorado Boulder, Boulder, CO United States of America; 3 Department of Physical Geography, Faculty of Geosciences, Utrecht University, Princetonlaan 8A, 3584 CB Utrecht, The Netherlands; 4 Department of Earth Sciences, Faculty of Geosciences, Utrecht University, Princetonlaan 8A, 3584 CB Utrecht, The Netherlands; 5 TNO Applied Geosciences, P.O. Box 80015, 3508 TA Utrecht, The Netherlands; 6 Present Address: Institute of Arctic and Alpine Research, University of Colorado Boulder, Boulder, CO United States of America; 7 Present Address: Department of Geography, Loughborough University, Loughborough, LE11 3TU United Kingdom; 8 Present Address: Deep-Time Consulting, Kroostweg 74, 3704 EG Zeist, The Netherlands

## Abstract

The high Arctic is the fastest warming region on Earth, evidenced by extreme near-surface temperature increase in non-summer seasons, recent rapid sea ice decline and permafrost melting since the early 1990’s. Understanding the impact of climate change on the sensitive Arctic ecosystem to climate change has so far been hampered by the lack of time-constrained, high-resolution records and by implicit climate data analyses. Here, we show evidence of sharp growth in freshwater green algae as well as distinct diatom assemblage changes since ~1995, retrieved from a high-Arctic (80 °N) lake sediment record on Barentsøya (Svalbard). The proxy record approaches an annual to biennial resolution. Combining remote sensing and *in-situ* climate data, we show that this ecological change is concurrent with, and is likely driven by, the atmospheric warming and a sharp decrease in the length of the sea ice covered period in the region, and throughout the Arctic. Moreover, this research demonstrates the value of palaeoclimate records in pristine environments for supporting and extending instrumental records. Our results reinforce and extend observations from other sites that the high Arctic has already undergone rapid ecological changes in response to on-going climate change, and will continue to do so in the future.

## Introduction

Over the past three decades, atmospheric warming has accelerated at a fast rate, disproportionally affecting the high Arctic. In the period 2000–2016, high Arctic (70–90°N) near-surface temperatures exceeded those of 1961–1990 by 1.8 °C^1,2^, almost 2.7 times more than the global mean. Atmospheric warming leads to, and is enhanced by, concurrent changes in other Arctic climate components^3^, most importantly sea ice loss. Less sea ice leads to a lower surface albedo, but also promotes heat trapping clouds and enhances atmospheric moisture^4^. Exceeding climate model predictions^5^, minimum (September) sea ice extent in the Arctic has decreased at a rate of 13.4% per decade^6^, with progressively thinner, perennial sea ice^7,8^.

A similar melt-albedo feedback exists on the scale of Arctic lakes: higher temperatures enhance spring snowmelt, melting snow cover and exposing the lake ice cover underneath earlier in summer. The darker ice absorbs a larger fraction of the solar radiation, which further promotes melting^9,10^. Additional controls on lake ice and snow cover are precipitation, which determines the thickness of the lake snow cover, and surface winds, which drive vertical mixing of the lake water column.

As Arctic lakes are still among the least impacted by human pollution^11^, they provide archives of biotic change in response to climate change without being obscured by other stressors^12^. The recent, rapid climate change in the Arctic has modified ecosystem functioning and caused ecosystem shifts in high-latitude lakes^12–15^. A longer ice-free season, extending the growing season for algae and other organisms, potentially leads to increased primary production and taxonomic shifts as new habitats become available^12,14,15^. As a result, ecological regimes may shift^16^ as climate related thresholds are exceeded^14^.

Yet, it is challenging to accurately determine the timing and geographical extent of ecological change in the high Arctic because of the generally low sedimentation rates and resulting low temporal resolution^12,14,15,17^, as well as an uneven distribution of studied locations^14^, e.g. resulting from inaccessibility of many high Arctic areas. In addition, ecological and climate change are generally only implicitly linked.

**Figure 1:**
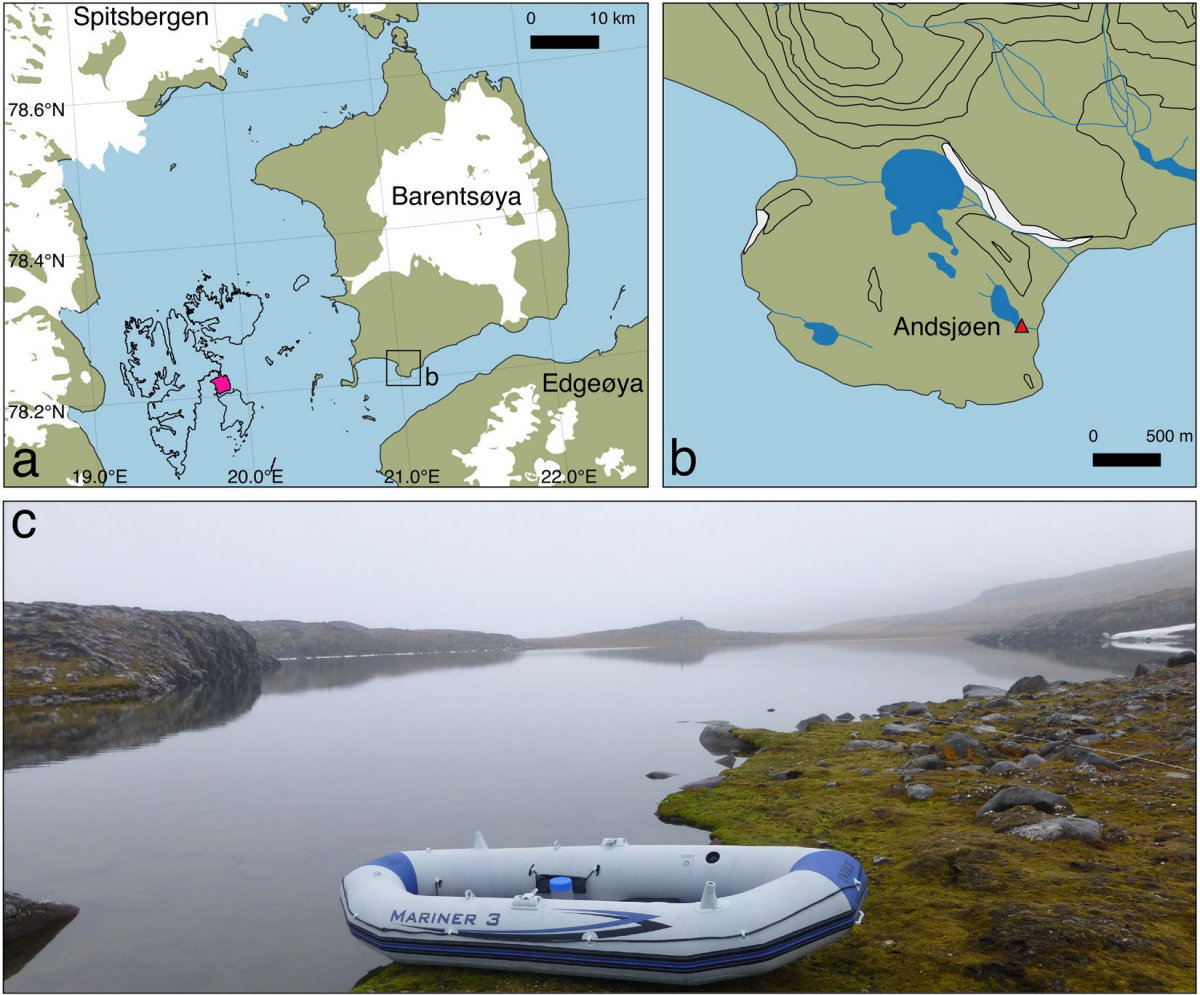
(**a**) Location of Barentsøya on the Svalbard archipelago (pink in inset). White areas are glaciers or ice caps, brown is tundra; (**b**) Zoom on southwest Barentsøya (Sundneset), and location of Andsjøen. Dark blue shows freshwater rivers/lakes. The maps in (**a**) and (**b**) were constructed using Quantum GIS (QGIS version 2.14); (**c**) Picture of Andsjøen taken on 25 August 2015 from the south towards the north (location shown by red triangle in (**b**)). Source for (**a**) and (**b**): Norwegian Polar Institute, http://geodata.npolar.no. The Norwegian Polar Institute’s free map products are licensed under the Creative Commons Attribution 4.0 International (CC BY 4.0) license, ©Norwegian Polar Institute.

Situated in the high Arctic, the Svalbard archipelago crosses several climate zones. West Svalbard is characterised by a remarkably mild climate for its near-polar location. The proximity of the relatively warm West Spitsbergen current prevents sea ice formation for most of the year. Towards the East and North, the Atlantic waters lose influence, and East Svalbard climate is increasingly controlled by the Barents Sea. Annual mean temperatures on East Spitsbergen, Edgeøya, and Barentsøya (Fig. 1) are about 4–8 °C lower than on the western coasts^18^, and sea ice coverage in Storfjorden and Freemansundet is much more extensive year-round.

By far the highest density of climate records is found on the more accessible west coasts of Spitsbergen. All of these show a strong temperature increase since the 1990’s^19,20^, whereas precipitation only shows small upward trends with substantial observational uncertainties^21^. In the longest available temperature record on Svalbard (Longyearbyen since 1921^21^), all years since 1989 were warmer than the 20^th^ century average, with 2010–2015 annual mean temperatures ~3.5 °C higher. Warming is even more pronounced in non-summer seasons^18^, due to the earlier open water exposure in spring and delayed sea ice cover in autumn^22^. As a result of this ongoing warming, most Svalbard glaciers and ice caps show signs of substantial thinning^23^. In the array of past anomalously warm winter seasons, Svalbard has experienced rapid permafrost thawing^24^ and several extreme weather events, such as winter rainfall, landslides, and wet snow avalanches. Although no long-term observations are available in the east and north, atmospheric reanalyses suggest additional warming in these regions^18^.

The majority of ecological studies on Svalbard, which has focussed on lakes in the western region, suggests that western Svalbard has undergone ecological regime shifts under changing climate^12,25,26^ in line with lakes in other Arctic regions^14^. Results from Edgeøya and Barentsøya in East Svalbard are scarce, not only for accessibility reasons, but also because the geology, consisting of mainly easily erodible sedimentary rocks, led to the absence of suitable lake basins.

Here we present a rare, ~100 year long East Svalbard sediment record from a small coastal lake (maximum 3.3 m water depth, ~375 m long (north-south), ~150 m wide (west-east)). Ecological change throughout the record was derived from *Pediastrum* (green algae) and pollen concentrations, and from *Pediastrum* and diatom percent relative abundance data. While *Pediastrum* and pollen data approach an annual to biennial resolution, the resolution of the diatom data is lower.

The combined use of diatom valves and *Pediastrum* enables the reconstruction of past water quality, habitat, catchment processes and lacustrine ecosystems^11^, providing complementary insight in ecological response to high Arctic climate change. Our freshwater ecological record is the first on remote East Svalbard, where direct anthropogenic impact is negligible. East Svalbard climate is colder than that of West Svalbard, and therefore more typical for the high Arctic as a whole. Furthermore, we link our ecological records to temperature time series reconstructed from nearby meteorological stations and to sea ice cover data retrieved through remote sensing to resolve any link between climate and ecological change.

## Results

### Ecological records

In August 2015, a 47 cm long sediment core was recovered from a small, shallow coastal lake (Andsjøen, Fig. 1) on southwest Barentsøya (78°12′N, 21°1′E, 15 m asl), located <100 m from the sea (Fig. 1c, see Methods). From this core, diatom, *Pediastrum* and pollen data were obtained from the past ~100 years. *Pediastrum* and pollen concentrations as well as percent relative abundance data of diatoms, *Pediastrum* and pollen have been used. The proxy record approaches an annual to biennial resolution (see Methods). Chronology is based on radiocarbon dating (^14^C) and on ^210^Pb, ^226^Ra and ^137^Cs isotopes (Bq/kg) (see Methods and Fig. S1).

**Figure 2:**
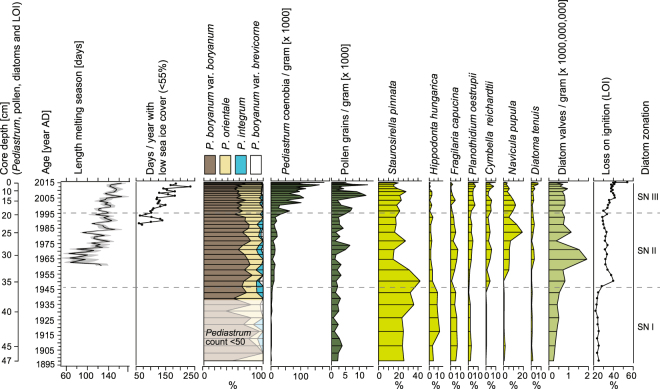
Synthesis of (approximate) melting season, sea ice cover near the studied site, and ecological data (*Pediastrum*, pollen and diatom valve concentrations, *Pediastrum* and diatom percent relative abundance data, and LOI) as derived from the Andsjøen core sediments. See text and Methods for a more detailed description on how the age model and individual variables were derived. Note that the lowermost sediment samples contained low *Pediastrum* concentrations, resulting in <50 *Pediastrum* coenobia per counted slide. This may have influenced the calculated *Pediastrum* percentages in this part of the core.

The diatom record (see Fig. 2 for the most abundant or dominant taxa) shows that the lake is dominated by a benthic community, with an absence of planktonic species, which is to be expected in a shallow lake with a maximum water depth of 3.3 m. Diatom concentrations fluctuate around 500 million to 1 billion valves per gram, without displaying a clear trend. *Staurosirella pinnata* (formally in the genus *Fragilaria*) dominates the assemblage, with ~20–40%. As a generalist, opportunistic species, *S. pinnata* can compete well under harsh conditions, such as prolonged ice cover, and is one of the most commonly observed species in the high Arctic in shallow lakes and ponds^15,25,27^. Two markedly significant biotic compositional changes are observed throughout the record, leading to a more complex assemblage. *Diatoma tenuis*, *Navicula pupula*, *Cymbella reichardtii* and *Planothidium oestrupii* show higher percentages from ~1950 onward, where *Diatoma tenuis* and *Planothidium oestrupii* profoundly increase from ~1995 onward. On the other hand, *Hippodonta hungarica* and *Fragilaria capucina* show a declining trend throughout the record (Supplementary Fig. S2).

Four *Pediastrum* species were identified from the core*: P. boryanum* var. *boryanum, P. orientale, P. integrum and P. boryanum* var. *brevicorne*. Together, *P. boryanum var. boryanum* and *P. orientale* make up for ~85–100% of the total *Pediastrum* sum, with *boryanum* var. *boryanum* being the dominant taxon throughout the record (Fig. 2). *P. boryanum* var. *boryanum* is a taxon associated with a wide range of eutrophic but not very polluted slightly alkaline freshwaters^28^. Yet, throughout the record, from ~1900 to 2015, a gradual trend towards higher *P. orientale* percentages can be observed. *P. orientale* is associated with clear water biotopes in distant regions^28^. Where diatom concentrations show no clear trend throughout the record, a gradual, tenfold increase in total *Pediastrum* concentrations (~2000 to ~20,000 *Pediastrum* coenobia per gram) is observed from ~1900 to ~1995. From 1995 until 2015, *Pediastrum* concentrations increase even more rapidly, showing an additional tenfold increase, towards ~200,000 coenobia per gram. Moreover, *Pediastrum* concentrations display a more erratic pattern from ~1995 onwards, with several outliers characterising the record (Fig. 2).

Pollen concentrations (pollen per gram) are low (Fig. 2), resulting in only few pollen per slide. As such, they do not allow for quantitative reconstructions of past vegetation dynamics. Yet, pollen concentrations appear to increase slightly from ~1995 until 2015 (Fig. 2).

Loss on ignition (LOI) values show a gradual increase in organic matter percentages of the sediments over the past century. Until ~1950, LOI is ~25 to 30%, between ~1950 and ~1995 LOI is ~35%. Until 2015, LOI values increase to ~40% (Fig. 2).

### Climate reconstruction

Located near the coast, local climate conditions at Andsjøen are strongly determined by the presence of sea ice. Here we use remote sensing sea ice data (see Methods) to show that sea ice cover around Svalbard shows signs of strong decline during the period 1979 to 2015. In the first ten years with full data coverage (1988–1997), while the ocean west of Svalbard was sea ice free for the entire year, sea ice cover over East Svalbard exceeded ~60% in most of the winter and spring (until Day of Year (DOY) 150, Supplementary Fig. S3). Ice cover lingered around in the inland sea between Spitsbergen in Barentsøya even during part of the summer, until regrowth started in early November. This changed distinctly since the late 1990’s and especially into the early 21^st^ century, when high sea ice coverage has been confined to late winter. Sea ice free season extends to both the spring, when sea ice disappears more quickly, and the autumn, when regrowth is delayed. This is confirmed when we take a regional average of the sea ice extent around the core location (Fig. 2). The sea ice free period (here arbitrarily taken as <55% cover, see Supplementary Figs S4 and S5), increases from 100 (1988–1997) to 183 days (2010–2014), with an approximately equal contribution of earlier spring melt (20 ± 4 days per decade earlier) and later sea ice growth (19 ± 4 days per decade later). The remote sensing data suggests that in the most recent years, sea ice no longer recovers in the winter: in 2010–2014, coverage exceeded 75% on ~17 days only, a decrease from 166 days in the 1988–1997 period. This is especially evident in very warm years such as 2007, 2010 and 2012 (Fig. 2, Supplementary Fig. S5).

Situated nearby the ocean, we expect that local temperature at the site increases in concert with the lengthening sea ice free season. In absence of long-term local temperature observations, we opted to reconstruct local temperature using a stacked Svalbard daily temperature record from 1960–2015, considering local climate conditions at the core site (see Methods). As a result of increasing air temperatures (0.7 ± 0.1 °C per decade, Supplementary Fig. S6), especially in spring and autumn, melting season length (see Methods) increases with 11 ± 1 days per decade in 1960–2015 (R^2^ = 0.57, p < 0.001). Ignoring local climate processes, such as the positive feedback loop between reduced ice coverage and temperature, as well as the stronger atmospheric warming in this region compared to the locations of the stations used in the reconstruction, this number should be considered a lower bound.

### Comparing ecological and climate data

Our rare sedimentary record that approaches an annual to biennial resolution allows for a direct comparison of climate and ecological data in the high Arctic. Our ecological records show that the second, most recent, shift in the benthic diatom record, towards a more complex diatom assemblage, from ~1995 AD to 2015, is concurrent with a tenfold increase in the concentrations of the planktonic algae *Pediastrum* and a slight increase in pollen concentrations. In this period, we also observe rising atmospheric temperatures, declining sea ice cover and a longer ice-free period.

## Discussion

In this study we combine ecological data from the high Arctic spanning the last century with climate observations. Since we have no daily temperature observations available for the time period before 1960, and no sea ice cover data before 1979, it is challenging to extend the direct comparison between climate and ecology further back in time. However, the reconstructed annual temperature time series from Longyearbyen^21^ (Supplementary Fig. S6) suggests that temperatures during the relatively warm 1930’s and 1940’s period were very similar to those from the 1990’s and early 2000’s. Yet, our results indicate that ecological changes in recent decades have been much more pronounced than during the 1930’s and 1940’s. A possible explanation is that the current warm period is much longer (since 1990) and is amplified in the most recent years: in the past decade, multiple record warm years have been observed since the start of the observations in 1912 (Supplementary Fig. S6). Under the expected continued human-induced global warming, which will be most intense in the high Arctic, it is not unlikely that ecological responses will be even more pronounced in the (near) future, as more non-reversible thresholds are likely to be exceeded.

A link between recent temperature, sea ice cover and changing lake ecology at Andsjøen is demonstrated in this study. However, this link is most likely indirect, and many potential drivers are directly or indirectly influenced by climate (e.g. prolonged growing season, reduced sea ice cover period, changed lake pH, lake conductivity, changed lake depth, etc.).

Not all of these drivers are equally likely to have caused the observed changes. Overall, the diatom taxa that increase throughout the record (*P. oestrupii, C. reichardtii* and *N. pupula*) are not particularly associated with a specific pH^29^, eliminating changed lake pH as the principal driver of the observed shifts. In addition, the absence of planktonic diatom taxa in the record suggests that the lake remained shallow throughout its deposition, indicating that lake level fluctuations are unlikely to have caused major changes in the diatom assemblage.

The more recent increase of *D. tenuis*, however, could be indicative of increased conductivity in the lake over recent years^27^. Similar increases have been observed in other shallow Arctic ponds and lakes in the Canadian high Arctic, and have been associated with increased evaporation related to the higher temperatures in the Arctic^15,30^. In addition, longer growing seasons, possibly the result of a reduced ice cover on the lake and overall higher temperatures, would create more habitats for various diatom taxa, for instance through the expansion of aquatic vegetation (e.g. moss). This would consequently result in a more ecologically complex and diverse diatom assemblage, like the assemblage observed in this study. A similar response of diatom assemblages to longer growing seasons was suggested by other studies in the Arctic^15,31,32^. Longer growing seasons would likely also affect ecology outside the lake by extending flowering periods, resulting in increased pollen concentrations in the lake sediments. We therefore conclude that the observed ecological patterns in this study are most likely driven by a combination of higher temperatures, reduced ice cover periods and longer growing seasons, affecting ecology through the mechanisms described above.

Besides climate change and climate change related drivers for ecological change, other mechanisms have been suggested to explain recent sudden ecological changes in other Arctic lakes. For instance, Holmgren *et al*.^25^ showed that atmospheric anthropogenic nitrogen deposition notably increased recently in four lakes in West Svalbard, consequently affecting the ecosystem. This increased nitrogen availability^25^, possibly aggravated by growing populations of birds in the catchment, like it is the case in West Svalbard^33^, could overshadow the true climate impact on pristine Arctic lakes such as Andsjøen. Yet, in contrast to studies on lakes in western Svalbard^25^, we do not observe signs for increased nitrogen availability in the lake. Unlike Holmgren *et al*.^25^, we do not observe a pronounced increase in diatom valves over recent years. Moreover, the diatom assemblage reflects oligotrophic to mesotrophic conditions^34,35^, and the taxa that increase throughout the record (*P. oestrupii, C. reichardtii* and *N. pupula*) are not associated with higher nutrient levels and thus eutrophication^25,34,35^. Finally, although *P. boryanum* var. *boryanum* dominates the record and is associated with eutrophic waters^28^, this species becomes less dominant in recent years. A gradual shift towards *P. orientale*, a species often associated with clear waters^28^, is even observed throughout the record. We therefore have no reason to assume that increased eutrophication or nitrogen availability in the lake played an important role in the observed recent ecological changes in Andsjøen the way it may have played a role in West Svalbard.

Similarly, sea ice variability and trends are not only governed by melt caused by global warming, but also partly controlled by large-scale sea ice drift which is driven by synoptic-scale atmospheric variability and oceanic currents. However, our hypothesis that melt dominates the sea ice variability near our site is supported by observations showing that recent sea ice trends around Svalbard are largely determined by local thinning (i.e. melt) instead of drift^36^. In addition, the sheltered location of our site protects it from strong winds, further limiting sea ice drift.

It should furthermore be noted that in comparison to diatoms, which have been widely used for reconstructing recent ecological change in the Arctic, existing work using *Pediastrum* is rare. Moreover, the existing *Pediastrum* work focuses mostly on shifts in *Pediastrum* assemblages^11^ rather than *Pediastrum* concentrations. Our study shows a sharp increase in *Pediastrum* concentrations that coincide with declining sea ice cover and a longer melting season. Although based on a single location, and although the observed increase in *Pediastrum* could partially be caused by diagenesis^37^ down-core, amplifying the trend of increasing *Pediastrum* toward the surface, this suggests that *Pediastrum* in high Arctic lakes directly responds to recent climate change. The increased *Pediastrum* productivity may even imply increased primary productivity in high Arctic lakes resulting from longer growing seasons caused by climate change, which was already suggested for sub-Arctic Canadian lakes^38^. *Pediastrum* may be a potential productivity indicator and an indicator for productivity and a longer growing season, but has so far not been used or recognised as such. Therefore, we recommend an increased focus on *Pediastrum* to support conclusions of recent studies^38,39^ that Arctic lake productivity increased over the past decades under influence of climate change.

Finally, although the temporal resolution of our study is much higher than that of most other high Arctic diatom studies, preventing a direct comparison of these records to our study and to recent climate changes, recent shifts in diatom assemblages can also be observed in other Arctic lakes^13,14,26,31,38^. This suggests climate-driven Arctic-wide ecological change occurred in recent years, regardless of the exact locality or remoteness of records or depth of the lakes. It furthermore confirms earlier realisations^14^ that even the most remote and northern ecosystems in the world are continuously being affected by human activities.

## Methods

### Sampling and site description

Fieldwork was conducted as part of the Netherlands Scientific Expedition Edgeøya Spitsbergen (SEES.NL) that took place from August 19 to 28, 2015. For the purpose of this study, a sediment core was collected on August 25, 2015 from the small lake Andsjøen, situated on Sundneset close to Freemansundet in the south east of Barentsøya (Fig. 1). Elevation of the lake is ~15 m above sea level, it is ~375 m long (north-south) and ~150 m wide (west-east). The lake is situated on a dolorite plateau and lacks inlets and outlets. After the deepest part of the lake had been determined by the use of a handheld sonar (depth = 3.3 m), a sediment core (47 cm) was retrieved from this location (78°12′28.9′′N, 21°03′27.8′′E) using a gravity corer deployed from a small inflatable boat (Fig. 1c). The sediment core was partitioned into 1 cm slices in the field in order to prevent mixing of the sediment.

### Chronology

Chronology is based on radiocarbon dating (^14^C) and on ^210^Pb, ^137^Cs and ^226^Ra isotopes.

For ^210^Pb, ^137^Cs and ^226^Ra dating, sediment samples from the core were analysed at the Royal NIOZ in the Netherlands using gamma-spectroscopy. The sample resolution for this analysis is ~5 cm. For each measurement, 2–5 ml of freeze-dried and homogenised sediment was weighed and packed in a 6-cm diameter plastic Petri dish which then was sealed with electrical insulation tape and packed in a sealed plastic envelope. Calibration and monitor standards and samples were prepared in the same geometry. Sealed sediment samples were left for a minimum of four weeks to establish radioactive equilibrium between ^222^Rn and ^226^Ra. Gamma activity was measured with a Canberra Broad Energy Range High Purity Germanium Detector (BEGe), using the 46.5 keV line for ^210^Pb, the 295.2, 351.9 and 609.3 keV lines for ^226^Ra and the 661.7 keV line for ^137^Cs. The detector, connected to a computer via a Digital Spectrum Analyser (DSA-1000), counted the radionuclide activities with Genie 2000 gamma spectroscopy software. Samples were counted for 2–5 days to obtain good counting statistics or relative standard deviation (RSD). A blank (empty petri dish) was counted during 23 days. The detector was externally calibrated with a Geological Certified Reference Material IAEA/RGU-1, with reference date of 01-01-1988. A monitor standard IAEA-300 provided quality control. Excess ^210^Pb activities were calculated by subtracting ^226^Ra activity averaged for the 295.2, 351.9 and 609.3 keV lines from the measured total ^210^Pb activity.

The CF or CRS (Constant Rate of ^210^Pb Supply) model was used to reconstruct ages from the measured ^210^Pb values, following recommendations by Sanchez-Carbeza and Fernández^40^ assuming that sedimentation rates potentially vary throughout the core. In addition, the maximum ^137^Cs concentration is assumed to correspond to 1962–1963 AD (e.g. conform Luoto *et al*.^33^) when testing of nuclear weapons reached its peak. This maximum was found between 24.5 and 35.5 cm depth, at around 29.5 cm depth. All isotope data can be found in Supplementary Dataset S1.

For AMS radiocarbon analysis, a *Salix polaris* leaf retrieved from 25.5 cm depth was analysed. AMS radiocarbon dating was carried out at the Centrum voor Isotopen Onderzoek (CIO) in Groningen, the Netherlands (GrA-67206). The radiocarbon activity correction (%) is 144.62 ± 4.4 and δ^13^C is −33.73, which corresponds to a Bomb-Peak calibrated age of 1962–1963 or 1971–1975 AD. Given the ^137^Cs peak at 29.5 cm depth and the ^210^Pb age reconstructions, we assume that the AMS radiocarbon date at 25.5 cm depth corresponds to 1971–1975 AD.

A second order polynomial was fitted through the reconstructed ages to obtain an age model for the whole core. The deepest part of the core has an approximate age of 1900 AD (47 cm depth). The age of the top of the core at the sediment-water interface is 2015. See Supplementary Fig. S1.

Sedimentation rates in the core increased from circa 0.03 cm/y around 1900 AD to about 0.2 to 0.3 cm/yr in the last decade (Fig. S7), which is high for a small Arctic lake with no inlets and outlets. The high sedimentation rate can be explained by the high moisture content in the sediments (65 to 95% near the top), making the sediments bulky.

### Pollen and *Pediastrum*

Samples were prepared for pollen analysis following the standard acetolysis procedure of Fægri and Iversen^41^ with the use of sodium polytungstate heavy liquid separation to remove clastic material. A known quantity of exotic spores (*Lycopodium clavatum*) was added to ~0.5 gram of sediment from each sample (~2,136 spores per sample) using a suspension based on *Lycopodium* tablets (~10,679 spores per tablet). The pollen residues were mounted in glycerine. Samples were analysed for pollen and *Pediastrum* under a light microscope with a magnification of 400x. Pollen types were identified following descriptions by Yao *et al*.^42^ and Moore *et al*.^43^.

Pollen concentrations were found to be low (mostly ~3000 to 4000 pollen grains per gram, resulting in ~20 pollen grains per slide). Pollen percentages are calculated on the basis of the total pollen sum, excluding pollen of aquatic plants. The record shows pollen representative of high-Arctic environments, such as *Salix* (Willow)*, Dryas, Saxifraga* (Saxifrages or rockfoils)*, Poaceae* (grasses) and *Caryophyllaceae* (Carnation family), but we also found high (up to 25%) percentages of allochtonous pollen of mostly *Pinus* (Pine tree). For these reasons, we decided to omit the pollen analysis in this study.

*Pediastrum* species are identified following Komárek & Jankovská^28^ and Turner *et al*.^44^, and are only counted if on estimation >50% of the coenobium is complete. Percentages of *Pediastrum* are based on the total sum of identified coenobia of *Pediastrum* species. *Pediastrum* and pollen was counted until a sum of ~300 *Pediastrum* coenobia was reached, or until ~100 *Lycopodium* spores were counted, or until the whole slide was counted. All pollen and *Pediastrum* data can be found in Supplementary Dataset S2.

### Organic content

LOI data provide information on the nature of the lithology of the Adsjøen sediments. The organic content (in weight percentage) of the samples taken from the core from the Andsjøen was determined by loss on ignition (LOI) following Heiri *et al*.^45^. Samples were taken from the core every cm of the 47 cm long core. The samples were dried (at 105 °C overnight to remove water from the sample) and ashed (4 h at 550 °C) to burn the organic matter from the sample. The weight difference between the weight of the ashed sample and the weight of the dried sample is the LOI (Supplementary Dataset S2).

### Diatoms

A total of 24 freeze dried samples with an average weight of 0.01 grams were processed with the water bath method^46^ using 30% hydrogen peroxide (H_2_O_2_) and 50% hydrochloric acid (HCl) to remove organic material and carbonates respectively. Permanent slides were prepared using Naphrax^TM^ as mounting medium and ≥300 valves were identified per slide under oil-immersion phase-contrast light microscope at 1000x magnification. A known quantity of microspheres was added for quantification of the concentration. All diatom data can be found in Supplementary Dataset S3. Significant zones were determined with the software package PSIMPOLL^47^ using the optimal splitting by information content method and the broken stick model. Zone boundaries are placed at approximately 1949 AD and 1994 AD. Diatom taxonomy is primarily based on Krammer and Lange-Bertalot^48–51^, Foged^52^ and Antoniades *et al*.^29^.

### Climate data

In this study we use remote sensing data of sea ice cover around Svalbard from the Oceans and Sea Ice Satellite Application Facility (OSI SAF). Covering the period from January 1979 to May 2015, this dataset uses passive microwave data (PMW) from the SMMR, SSM/I and SSMIS sensors. Ice concentration is computed from atmospherically corrected PMW brightness temperatures, using a combination of state-of-the-art algorithms and dynamic tie-points. Time resolution is daily, with partial coverage prior to 1987, and full coverage available after that time. The total uncertainty of the derived sea ice extent (which is default part of the algorithm output) in the area around Barentsøya is ~13%, which varies only marginally over the time period and throughout the year. The data are presented on a Lambert Azimuthal Equal Area polar projection, with a grid spacing of 25 km.

To reconstruct temperature and melting season at the Andsjøen site, where no weather observations are available, we use observed near-surface (2 meter) temperature series of five stations on or near Svalbard (Supplementary Table S1) with observations prior to 1980. Bjørnøya station was discarded from the analysis, because of its southern location and much higher mean temperature than at the other stations. The weather station located closest to the site (Kapp Heuglin) only started in 1992 and only has two years of complete data coverage (2007–2008). The data of the other stations, where annual mean temperature differs with less than 1 °C (−5.0 to −5.7 °C, Supplementary Table S1), are stacked and averaged to get a blended reconstruction of the 1960–2015 temperature (Supplementary Fig. S6). The difference in annual mean temperature between this blended dataset and the coring site location according to Van Pelt *et al*.^18^ (VP16 hereafter) (−1.6 °C, Table S1) is used to then reconstruct the temperature at the site: each daily temperature from the blended dataset is simply subtracted with −1.6 °C (Supplementary Table S1).

As an additional evaluation of our approach, we compared the reconstructed temperature at the core site with that of the Kapp Heuglin weather station for the two overlapping years. The difference in temperature is only 0.3 °C, which confirms the validity of our method. The associated uncertainty is defined as the mean difference between the observed temperature at the five stations and the VP16 temperature at these locations (0.5 °C, Supplementary Table S1). Finally, the length of the melting season (Fig. 2) is calculated as the annual number of days when daily maximum temperature exceeded the melting point.

### Data availability

Ecological and chronological data acquired in the project are found in the Supplementary Information (Supplementary Datasets S1, S2 and S3). The global temperature data from GISTEMP are available via https://data.giss.nasa.gov/gistemp/. Sea ice data are available on http://osisaf.met.no/. Temperature data are available through the Norwegian Meteorological Institute.

## Electronic supplementary material

Supplementary Information

Dataset S1

Dataset S2

Dataset S3
