# Supplementary material for: Recent climate warming drives ecological change in a remote high-Arctic lake
Source: Sci Rep. 2018 May 1;8:6858. doi: 10.1038/s41598-018-25148-7 (PMC5931553; doi:10.1038/s41598-018-25148-7)
Supplement: Supplementary file 1 — Supplementary Information [file 41598_2018_25148_MOESM1_ESM.docx]

**Supplementary Information for ‘Recent climate warming drives ecological change in a remote high-Arctic lake’**

**Authors:**

**Lineke Woelders**

**Jan T. M. Lenaerts**

**Kimberley Hagemans**

**Keechy Akkerman**

**Thomas B. van Hoof**

**Wim Z. Hoek**

*Datasets S1: Chronology data.*

*Dataset S2: Pediastrum, pollen and LOI data*

*Dataset S3: Diatom data*

**

*Figure S1: a. ^137^Cesium, ^226^Radon and ^210^Pb concentrations (Bq/kg) throughout the core. b. The Cesium peak at 1962-1963 AD (maximum ^137^Cs activity occurs between 24.5 and 35.5 cm depth, around 29.5 cm depth, see a.), the AMS ^14^C radiocarbon Bomb-Peak calibrated age at 1971-1975 and the ^210^Pb-based reconstructed ages derived using the CRS model. Dotted line is the derived age model from these individual reconstructed ages: a fitted second order polynomial (R^2^=0.98997).*

**

*Figure S2: Diatom concentrations and percentages of most abundant taxa (>5% of the diatom assemblage, at least in one sample)*

**

*Figure S3: Daily mean sea ice distribution around the Svalbard archipelago, averaged over the period 1988-1997 (left) and 2010 to 2014 (right). DOY=Day of Year. The maps were constructed using NCAR’s Command Language (NCL version 6.4, doi: http://dx.doi.org/10.5065/D6WD3XH5)*

**

*Figure S4: Time series (1987-2014) of annual number of days with the regional sea ice extent below given threshold*

**

*Figure S5: Daily, regional mean sea ice concentration around core site (20.5-21°E, 78-78.5°N) for 1978-2015. The left and right dashed red line illustrate the earliest and latest day of sea ice concentration <55%, respectively. Grey colors denote missing data. The high fraction of days with missing data prior to 1987, prevent us from calculating the number of days below a sea ice threshold (Figure 2, Figure S4) in that period*

**

*Figure S6: Time series (1912-2016) of annual mean temperature at five Svalbard weather stations (coloured lines, see Table S1 for station details), and the reconstructed annual mean temperature at the core site (black line)*

*Figure S7: Reconstructed sediment accumulation rates in the sediment core*

**Table S1: Observed and VP16 (see Methods) temperatures from several stations nearby Andsjøen**

| Station name | Latitude (°N) | Longitude (°E) | Elevation (m asl) | Time period | Mean observed temperature (°C) | VP16 temperature |
| --- | --- | --- | --- | --- | --- | --- |
| Bjørnøya (BJ) | 74.52 | 19.01 | 16 | 1960-2015 | -1.5 | - |
| Hopen (HO) | 76.51 | 25.01 | 6 | 1960-2015 | -5.2 | -5.3 |
| Longyearbyen (LO) | 78.25 | 15.51 | 28 | 1975-2015 | -5.0 | -5.7 |
| Ny-Ålesund (NY) | 78.92 | 11.93 | 8 | 1974-2015 | -5.0 | -5.9 |
| Sveagruva (SV) | 77.88 | 16.72 | 9 | 1978-2015 | -5.7 | -6.0 |
| ***Andsjøen (core)*** | ***78.21*** | ***21.06*** | ***15*** | ***1960-2015*** | ***-*** | ***-7.3 (reconstr.)*** |
| Kapp Heuglin | 78.25 | 22.82 | 14 | 2007-2008 | -6.2 |  |
| *Andsjøen (core)* | *78.21* | *21.06* | *15* | *2007-2008* | - | *-5.9 (reconstr.)* |
